# Decreased Susceptibility of *Shigella* Isolates to Azithromycin in Children in Tehran, Iran

**DOI:** 10.1155/2022/4503964

**Published:** 2022-03-27

**Authors:** Parisa Behruznia, Mehrzad Sadredinamin, Ali Hashemi, Bahareh Hajikhani, Neda Yousefi Nojookambari, Mahboobeh Behruznia, Zohreh Ghalavand

**Affiliations:** ^1^Department of Microbiology, School of Medicine, Shahid Beheshti University of Medical Science, Tehran, Iran; ^2^Division of Ecology and Evolution, Research School of Biology, The Australian National University, Canberra, Acton 2601, Australia

## Abstract

Azithromycin (AZT) has widely been used for the treatment of shigellosis in children. Recent studies showed a high rate of decreased susceptibility to azithromycin due to different mechanisms of resistance in *Shigella* isolates. Accordingly, the purpose of this study was to investigate the role of azithromycin resistance mechanisms of *Shigella* isolates in Iran during a two-year period. In this study, we investigated the mechanisms of resistance among *Shigella* spp. that were isolated from children with shigellosis. The minimum inhibitory concentration (MIC) of *Shigella* isolates to azithromycin was determined by the agar dilution method in the presence and absence of Phe-Arg-*β*-naphthylamide inhibitor. The presence of 12 macrolide resistance genes was investigated for all isolates by PCR for the first time in Tehran province in Iran. Among the 120 *Shigella* spp., only the *mph(A)* gene (49.2%) was detected and other macrolide resistance genes were absent. The phenotypic activity of efflux pump was observed in 1.9% of isolates which were associated with over expression of both *omp(A)* and *omp(W)* genes. The high prevalence of the *mph(A)* gene among DSA isolates may indicate that azithromycin resistance has evolved as a result of antimicrobial selection pressures and inappropriate use of azithromycin.

## 1. Introduction

*Shigella* species are Gram-negative and nonmotile rods in the family Enterobacteriaceae that cause shigellosis. Shigellosis was the third leading cause of diarrheal death in children under the age of 5 in 2015 (40,000 deaths per year) [1–3], and it mostly affects children living in developing countries [4–6]. *Shigella* can be classified into four serogroups or species based on O lipopolysaccharide antigen type: *S. dysenteriae* (subgroup A), *S. flexneri* (subgroup B), *S. boydii* (subgroup C), and *S. sonnei* (subgroup D) [5, 7]. Based on epidemiological studies, *S. flexneri* and *S. sonnei* are the most common species in developing countries, but *S. sonnei* is the predominant species in developed countries [5, 8–10]. Recent studies have revealed a species shift from *S. flexneri* to *S. sonnei* in Iran, and *S. sonnei* has been the dominant species in most parts of the country [11–14]. *Shigella* spp. are highly infectious and are transmitted by the fecal-oral route or ingestion of contaminated food or water [5, 8, 15]. While shigellosis is endemic in developing countries with poor water and sanitation conditions, it is usually associated with either returned travelers or men who have sex with men (MSM) in developing countries (3–5). Shigellosis is transmitted in the developing countries as fecal-oral and contaminated food and water, and in developed countries, it is transmitted by traveling to disease-endemic regions and men who having sex with men [8, 10, 16]. Symptoms appear abruptly after an incubation period of 12 hours to approximately 2 days and include high fever, crampy abdominal pain, and diarrhea [17]. The disease is self-limiting, but antibiotic treatment is required in children, elderly, and people with weakened immune systems [18].

*Shigella* spp. have become resistant to first-line drugs (trimethoprim-sulfamethoxazole and ampicillin) and are no longer prescribed to treat shigellosis due to the emergence of multidrug resistance and has challenged the treatment of the disease in children [4, 5, 19, 20]. These drugs are replaced by ciprofloxacin (CIP), and azithromycin (AZT) for shigellosis treatment in adults and children [4, 5, 21]. Owing to its oral administration and affordability, AZT is recommended by a number of international guidelines for the treatment of shigellosis in children [4]. In Iran, AZT is the most commonly prescribed antibiotic for treatment of children suffering from shigellosis [14, 22]. Reports of *Shigella* isolates with decreased susceptibility to azithromycin (DSA) are increasing globally, raising concerns about its usefulness as the second-line treatment for children with shigellosis [4, 23]. The most common types of macrolide resistance in *Enterobacteriaceae* are those encoded in mobile genetic elements, such as target site modification by methylases encoded by *erm* genes (*erm(A)*, *erm(B)*, *erm(C)*, *erm(F)*, *erm(T)*, *erm(X)*), inactivation of macrolides, mediated by esterases such as those encoded by *ere* genes (*ereA* and *ereB*), and phosphotransferases encoded by *mph* genes (*mph(A)* and *mph(B)*). Additionally, the macrolide efflux pumps encoded by *mef* genes (*mef(A)* or *mef(B) and msr(A))* and chromosomal efflux pumps (*omp(A)* and *omp(W)*) have been reported to confer resistance to macrolides [24]. To a lesser extent, mutations in the L4 (*rplD*) and L22 (*rplV*) ribosomal proteins and in 23S rRNA (*rrlH*) have been shown to be responsible for macrolide resistance [24, 25].

Recent studies have reported a relatively high frequency of resistance to azithromycin among *Shigella* isolates from children with dysentery in Iran [12, 14, 26]. There has been no detailed investigation of the mechanisms of macrolides resistance among the DSA-*Shigella* isolates in Iran. In this study, we determined the azithromycin MICs for a collection of *Shigella* isolates recovered from children with shigellosis in Tehran, Iran. Then, we investigated the presence of macrolide resistance genes associated with mobile genetic elements and the expression levels of outer membrane proteins A and W (*ompA* and *ompW*) genes related to efflux pump in isolates.

## 2. Materials and Methods

### 2.1. Bacterial Isolates and Identification

**Figure 1:**
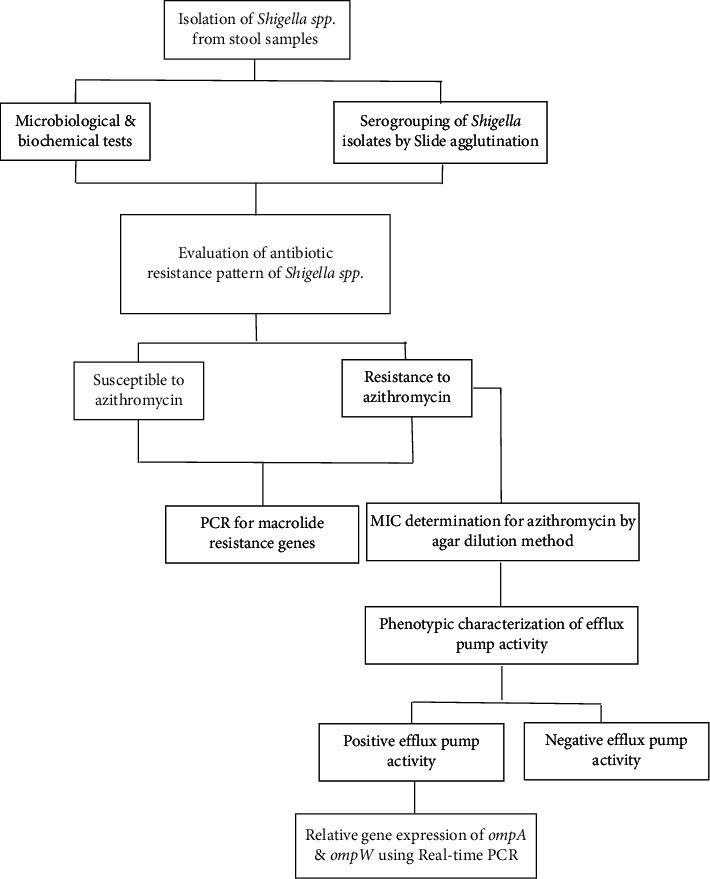
Work flowchart for identification *Shigella* isolates with decreased susceptibility to azithromycin (DSA) and characterization of the related genetic mechanisms.

*Shigella* isolates were collected between March 2017 and September 2019 from the feces of children under 14 who were suspected to have shigellosis and were referred to Children's Medical Center in Tehran. Initial identification was performed using microbiological and biochemical analysis and *Shigella* serogroups were determined using latex agglutination serotyping (Figure 1). This study was evaluated by the Local Ethics Committee of Shahid Beheshti University of Medical Sciences (IR.SBMU.MSP.REC.1399.490).

### 2.2. Antibiotic Susceptibility Test and MICs of Azithromycin

The antibiotic susceptibility pattern of all isolates had been previously described [12]. Briefly, antimicrobial susceptibility testing to nine antibiotics was conducted using Kirby–Bauer disk diffusion method. The MICs of DSA isolates were confirmed (ranging from 2 to 512 *µ*g/ml) using the agar dilution method according to the Clinical and Laboratory Standards Institute (CLSI) guidelines (Clinical and Laboratory Standards Institute (CLSI), Performance standards for antimicrobial susceptibility testing, 29th ed., CLSI supplement M100–S29, Wayne, PA; [27]).

### 2.3. MICs of Azithromycin in the Presence of Efflux Pump Inhibitor

The MICs of DSA isolates were examined by adding efflux pump inhibitor Phe-Arg-*β*-naphthylamide (PA*β*N) (20 mg/ml) (Sigma, St. Louis, Mo., USA) to determine the impact of efflux pumps activity on azithromycin resistance. *A* ≥4-fold reduction in azithromycin MIC in the presence of PA*β*N suggested the existence of an efflux pump (27, 28).

### 2.4. The Presence of Macrolide Resistance Genes

Genomic DNA was extracted using the High Pure Isolation Kit (Roche, Mannheim, Germany) according to the manufacturer's instructions. Macrolide resistance genes, including, *mph(A)*, *mph(B)*, *erm(A)*, *erm(B)*, *erm(C)*, *erm(F)*, *erm(T)*, *erm(X)*, *ere(A)*, *ere(B)*, *mef(A)*, and *msr(A)* were amplified by polymerase chain reaction (PCR) using specific primers (Table 1). PCR products were separated using 1.5% agarose gel. Positive PCR products were sequenced (Stab vida, Spain) to confirm the presence of resistance genes (accession number: OL310860).

### 2.5. Quantitative Real-Time PCR (qRT-PCR) for Evaluation of Efflux Pumps Genes Expression

Total RNA was extracted using a BioFACT TM Total RNA Prep Kit (Biofact, South Korea) following the manufacturer's instructions. All extracted RNAs were treated with DNase I (CinnaGen Co., Iran) in order to remove the remaining genomic DNA. Reverse transcription was performed using the Add Script cDNA Synthesis Kit (Add bio, South Korea) with an input of 200 ng/*µ*l of total RNA in a final reaction volume of 20 *µ*L under standard reverse transcription PCR conditions following the manufacturer's instructions.

The expression level of efflux pump genes among phenotypically active isolates was determined by quantitative real-time-PCR (qRT-PCR) using primers targeting *omp(A)* and *omp(W)* genes as described previously [30] (Table 1). All reactions were conducted in duplicate, and the 16S rRNA was used as the endogenous control gene. The 2^−ΔΔCT^ method was used to determine the relative expression of the target genes, and a value of ≥4-fold compared to that of *S. flexneri* ATCC12022 was considered as overexpression [31].

### 2.6. Statistical Analysis

Pearson's chi-squared test was used to investigate the relationship between antibiotic resistances with regard to the age group of the patients, gender and the species of *Shigella* isolated from the patients. A *p* value of <0.05 was considered significant. Data were analyzed using JMP, version16 (SAS Institute Inc., 2021).

## 3. Results

### 3.1. Characteristics of the Patients and Isolates

A total of 120 *Shigella* isolates were collected from the fecal samples of children with shigellosis Sixty percent of patients were male (*n* = 72), and 40% were female (*n* = 48) (Table 2). Overall, 55% of patients (*n* = 66) aged 5 years old or younger, 35% (*n* = 42) aged 6 to 10, and 10% (*n* = 12) aged 11 to 14 years old. Among 120 *Shigella* isolates, *S. sonnei* was the most common species with 80.8% of the total isolates (*n* = 97), followed by *S. flexneri* with 17.5% (*n* = 21) and *S. boydii* with 1.7% (*n* = 2), respectively. The type of *Shigella* spp. detected in a patient did not vary with respect to age group and gender of the patients (*p* > 0.05).

The azithromycin MICs among the *S. sonnei* isolates ranged from 32 to 512 *µ*g/ml, and the only *S. flexneri* isolates had MIC = 32 *µ*g/ml. Of the 54 DSA-*Shigella* isolates, only one isolate (1.9%) was *S. flexneri*, and the other 53 isolates (98.1%) were *S. sonnei*. All DSA isolates were resistant to Trimethoprim/sulfamethoxazole. A high frequency of isolates was resistant to ampicillin (96.2%), nalidixic acid (94.4%), cefotaxime (90.7%), cefixime (90.7%), and minocycline (79.6%). The frequency of resistance to ciprofloxacin and levofloxacin was comparatively low and was 3.7% and 16.6%, respectively. The probability of detecting DSA isolates varied with respect to the age group of the patients (*p* < 0.05), and children between 11 and 14 years old showed a higher prevalence of DSA isolates. However, the probability of detecting DSA-*Shigella* isolates did not vary with regard to the gender of the patients (*p* > 0.05).

### 3.2. Identification of Efflux Pump-Mediated Resistance

**Figure 2:**
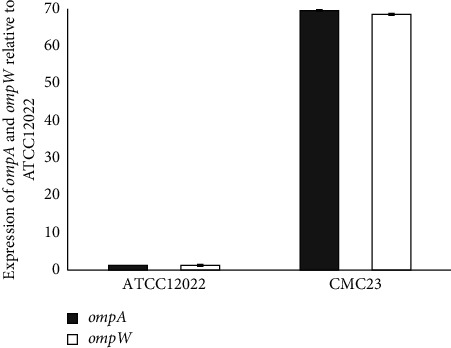
Relative expression of *ompA* and *ompW* genes by qRT-PCR. The relative expression level after being normalized to the expression of the reference gene 16S rRNA was compared relative to that in *S. flexneri* ATCC 12022. Results shown are the mean ± std of two independent experiments.

All DSA-*Shigella* isolates were able to grow in the presence of PA*β*N. Overall, MIC levels of 8 isolates (14.8%) decreased in the presence of PA*β*N, irrespective of the initial MIC of azithromycin (MICs ranging from 32 to 256 *µ*g/ml). Only one *S. sonnei* isolate demonstrated a decrease of ≥4-fold in azithromycin MIC when PA*β*N was added. The MIC of this isolate dropped from 512 *µ*g/ml to 64 *µ*g/ml in the presence of the inhibitor. The qRT-PCR results also showed overexpression in the mRNA levels of efflux pump genes *ompA* and *ompW* (Figure 2). However, mRNA overexpression was not detected among the other isolates that were inhibited by PA*β*N.

### 3.3. Characterization of Macrolide Resistance Genes

**Figure 3:**
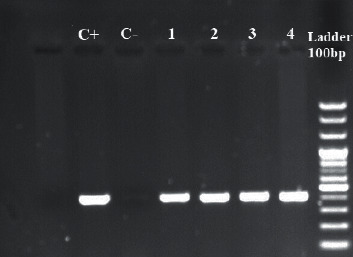
PCR products of *mphA* gene among *Shigella* clinical strains. Lane 2, positive control (*S. Sonnei* accession number: OL310860); lane 3, negative control; and lane 4, 5, 6, and 7 including sample 1 to 4, *Shigella* isolates harboring *mphA* gene.

The PCR analysis demonstrated that 59 (49.2%) of *Shigella* isolates carried the *mph(A)* gene. Five isolates that were susceptible to azithromycin (MICs <16) were found to carry the *mph(A)* gene. However, other mobile genetic elements associated with the azithromycin resistance (including *mph(B)*, *erm(A)*, *erm(B)*, *erm(C)*, *erm(F)*, *erm(T)*, *erm(X)*, *ere(A)*, *ere(B)*, *mef(A)*, and *msr(A)* were not detected among these isolates (Figure 3). Of 59 isolates *mph(A)* positive, 32 (54.2%) exhibited high levels of resistance to azithromycin (MICs ≥64 *µ*g/ml). Demographics and clinical features of pediatric patients are accessible in detail in Table S1.

## 4. Discussion

In the collection of 120 *Shigella* isolates in this study, 54 isolates (45%) were confirmed to be DSA, indicating more considerations should be taken in prescribing this drug for the treatment of children with shigellosis. Azithromycin MICs of the DSA-*Shigella* isolates ranged from 32 to 512 *µ*g/ml, and 59.2% of the isolates demonstrated high levels of resistance to azithromycin (MICs ≥64 *µ*g/ml). Previous studies have also reported a relatively high frequency of azithromycin resistance in *Shigella* isolates. For example, the rate of DSA was 42% in Palestine [32], 20.4% in China [31], 20% in the US [33], 13% in Australia [34], and 5% in Southeast Asia [4]. The lower rate of DSA reported from Southeast Asia has been associated with limited azithromycin usage in the region [4]. A previous study by Ezernitchi et al. [1] found that DSA-*Shigella* isolates were obtained mainly from children under 9 years of age. The results of our study showed that the age of the patients had a significant impact on the prevalence of DSA isolates among children with shigellosis. The 11- to 14-years-old patients were more likely to harbor DSA-*Shigella* even though this age group represented only 10% of cases with shigellosis.

Previous studies have established the importance of acquired mobile genetic elements in conferring resistance to macrolides in *Shigella* and other Enterobacteriaceae [24, 25, 31]. We investigated the presence of 12 mobile genetic elements associated with azithromycin resistance, and 49.2% (59/120) of the isolates were positive for the *mph(A)* gene. However, other mobile genetic elements associated with the azithromycin resistance were not detected in our *Shigella* isolates. Five isolates (8.5%) were found to carry the *mph(A)* gene, but they were susceptible to azithromycin (MICs <16). This heterogeneity could be explained by the differential expression levels in individual cells due to the variation in *mph(A)* copy numbers, leading to differences in azithromycin resistance levels [24]. Overall, this finding is consistent with that of the previous studies, which reported the role of the *mph(A)* gene as the principal mechanism for azithromycin resistance in *Shigella* isolates [4, 29]. For example, Zhang et al. [31] found that 55% of DSA-*Shigella* isolates were *mph(A)* positive, and no other resistance gene was detected. Liu et al. [29] reported that 57.8% and 40.7% of *S. flexneri* and *S. sonnei* isolates carried the *mph(A)* gene, but other azithromycin resistance genes were not detected. A very low frequency (0.6%) of DSA-*Shigella* from Southeast Asia were positive for the *erm(B)* gene [4]. Likewise, *erm(B)*-associated azithromycin resistance was detected in 3.4% of the *E. coli* isolates with DSA in Peru [24].

PA*β*N is an efflux pump inhibitor, which competes with macrolides for its specific binding point. The role of PA*β*N-inhibitable efflux pumps in azithromycin resistance has been demonstrated in *Shigella* spp. and *E. coli* [24, 30]. In this study, one *S. sonnei* isolate (1.9%) demonstrated azithromycin resistance associated with the efflux pump activity. This isolate contained *omp(A)*, *omp(W)* and *mph(A)* genes. Several studies have reported that mutations in the ribosomal proteins L4 (*rplD*) and ribosomal proteins L22 (*rplV*) and in 23S rRNA (*rrlH*) can confer macrolide resistance [24, 35]. Unfortunately, we did not determine the nucleotide sequence changes of the specific regions of the three genes, and we could not determine the azithromycin resistance mechanism in one *S. sonnei* isolate. Further studies are required to understand the possible additional mechanisms responsible for the DSA in *Shigella* spp. The present study demonstrated that the plasmid-mediated *mph(A)* gene is the most common macrolide resistance gene in *Shigella* isolates collected from children with shigellosis in Tehran, Iran. The high prevalence of the *mph(A)* gene among DSA isolates may indicate that azithromycin resistance has evolved as a result of antimicrobial selection pressures and inappropriate use of azithromycin. The plasmid-mediated *mph(A*) gene can spread quickly among different members of the *Enterobacteriaceae* and yield either the same or different strains with DSA [36].

## 5. Conclusion

Contrary to most studies, which have shown that efflux pump has no role in azithromycin resistance, our study showed that one of our DSA isolates increased *omp(A)* and *omp(W)* expression levels and consequently, efflux pump can play a role in resistance.

## Figures and Tables

**Table 1:** Primer sequences.

Target gene	Primer sequence (5′ ⟶ 3′)	Product size (bp)	Annealing temperature (°C)	Reference
*mph(A)*	F: GTGAGGAGGAGCTTCGCGAG	403	59	Rahman et al. [28]
	R: TGCCGCAGGACTCGGAGGTC			
*mph(B)*	F: GATATTAAACAAGTAATCAGAATAG	494	49	Rahman et al. [28]
	R: GCTCTTACTGCATCCATACG			
*erm(A)*	F: TCTAAAAAGCATGTAAAAGAAA	533	47	Rahman et al. [28]
	R: CGATACTTTTTGTAGTCCTTC			
*erm(B)*	F: GAAAAAGTACTCAACCAAATA	639	43	Rahman et al. [28]
	R: AATTTAAGTACCGTTACT			
*erm(C)*	F: TCAAAACATAATATAGATAAA	642	43	Rahman et al. [28]
	R: GCTAATATTGTTTAAATCGTCAAT			
*erm(F)*	F: CGACACAGCTTTGGTTGAAC	302	52	Liu et al. [29]
	R: GGACCTACCTCATAGACAAG			
*erm(T)*	F: CATATAAATGAAATTTTGAG	369	42	Liu et al. [29]
	R: ACGATTTGTATTTAGCAACC			
*erm(X)*	F: GAGATCGGRCCAGGAAGC	488	54	Liu et al. [29]
	R: GTGTGCACCATCGCCTGA			
*ere(A)*	F: GCCGGTGCTCATGAACTTGAG	420	55	Rahman et al. [28]
	R: CGACTCTATTCGATCAGAGGC			
*ere(B)*	F: TTGGAGATACCCAGATTGTAG	537	50	Rahman et al. [28]
	R: GAGCCATAGCTTCAACGC			
*mef(A)*	F: AGTATCATTAATCACTAGTGC	345	48	Rahman et al. [28]
	R: TTCTTCTGGTACTAAAAGTGG			
*msr(A)*	F: GCACTTATTGGGGGTAATGG	384	51	Rahman et al. [28]
	R: GTCTATAAGTGCTCTATCGTG			
*omp(A)*	F: ATGAAAAAGACAGCTATCGCG	187	54	Gomes et al. [30]
	R: CACCAAAAGCACCAGCGCCCA			
*omp(W)*	F: GTTAACAGTGGCGGGTTTGGC	154	56	Gomes et al. [30]
	R: CACGCTGAATCCACCCAGACT			

bp, base pair; F, forward primer; R, reverse primer.

**Table 2:** Characteristics of the patients with shigellosis.

Age group	Gender no.	*Shigella* spp.		
		*S. sonnei* no.	*S. flexneri* no.	*S. boydii* no.
≥5	Male: 46	37	8	1
	Female: 20	15	4	1
	Total: 66	52	12	2
6–10	Male: 16	15	1	0
	Female: 26	22	4	0
	Total: 42	37	5	0
11–14	Male: 10	7	3	0
	Female: 2	1	1	0
	Total: 12	8	4	0
Total	Male: 72	59	12	1
	Female: 48	38	9	1
	Total: 120	97	21	2

No.: number.

## Data Availability

All the data generated or analyzed during this study were included in this article.

## Supplementary Materials

Supplementary Materials Table S1: clinical data of pediatric patients, drug resistance, and distribution of azithromycin resistance genes in DSA-*Shigella* spp.
