# Corrigendum to “Decreased Susceptibility of *Shigella* Isolates to Azithromycin in Children in Tehran, Iran”

**DOI:** 10.1155/cjid/9894508

**Published:** 2025-06-04

P. Behruznia, M. Sadredinamin, A. Hashemi, et al., “Decreased Susceptibility of *Shigella* Isolates to Azithromycin in Children in Tehran, Iran,” *Canadian Journal of Infectious Diseases and Medical Microbiology* 2022, no. 1 (2022): 1–7, https://doi.org/10.1155/2022/4503964.

In the article titled “Decreased Susceptibility of *Shigella* Isolates to Azithromycin in Children in Tehran, Iran,” there was an error in affiliation number 1. In the corrected affiliation, the “Shahid Beheshti University of Medical Science” should be changed to “Shahid Beheshti University of Medical Sciences” in the article. The corrected affiliation appears below:

“*Department of Microbiology, School of Medicine, Shahid Beheshti University of Medical Sciences, Tehran, Iran*”.

We apologize for this error.

