# Supplementary material for: Decreased Susceptibility of Shigella Isolates to Azithromycin in Children in Tehran, Iran
Source: Can J Infect Dis Med Microbiol. 2022 Mar 27;2022:4503964. doi: 10.1155/2022/4503964 (PMC8977332; doi:10.1155/2022/4503964)
Supplement: Supplementary Materials — Table S1: clinical data of pediatric patients, drug resistance, and distribution of azithromycin resistance genes in DSA-Shigella spp. [file 4503964.f1.docx]

| Table S1. Cont | | | | | | | | | |
| --- | --- | --- | --- | --- | --- | --- | --- | --- | --- |
|  | P4 | P6 | P11 | P13 | P17 | P23 | P29 | P32 | P48 |
| Age | Under 5 years | Under 5 years | 6- 10 years | 6 - 10 years | Under 5 years | 11 - 14 years | Under 5 years | 11 - 14 years | Under 5 years |
| Gender | Male | Male | Female | Female | Male | Female | Male | Male | Female |
| Ward | OPD | OPD | OPD | OPD | OPD | Surgery | Immunology and rheumatology | Emergency | Emergency |
| Resistance pattern | NA/AMP/SXT/AZM | CTX/NA/AMP/SXT/CFM/AZM | CTX/NA/AMP/SXT/CFM/AZM | CTX/NA/AMP/SXT/CFM/AZM | CTX/NA/AMP/SXT/CFM/AZM | CTX/NA/AMP/SXT/CFM/AZM | CTX/NA/AMP/SXT/CFM/AZM | NA/AMP/SXT/AZM/MN | NA /SXT/AZM/MN |
| *mphA* | No | No | No | No | No | No | No | No | No |
| *mphB* | No | No | No | No | No | No | No | No | No |
| *ermA* | No | No | No | No | No | No | No | No | No |
| *ermB* | No | No | No | No | No | No | No | No | No |
| *ermC* | No | No | No | No | No | No | No | No | No |
| *ermF* | No | No | No | No | No | No | No | No | No |
| *ermX* | No | No | No | No | No | No | No | No | No |
| *ermT* | No | No | No | No | No | No | No | No | No |
| *ereA* | No | No | No | No | No | No | No | No | No |
| *ereB* | No | No | No | No | No | No | No | No | No |
| *mefA* | No | No | No | No | No | No | No | No | No |
| *msrA* | No | No | No | No | No | No | No | No | No |
| Azithromycin MIC (µg/ml) in the presence of inhibitor | 512 | 64 | 64 | 64 | 64 | 128 | 64 | 512 | 16 |
| Azithromycin MIC(µg/ml) in the absence of inhibitor | 512 | 64 | 64 | 64 | 64 | 512 | 64 | 512 | 32 |

Table S1. Clinical data of pediatric patients, drug resistance and distribution of azithromycin resistance genes in DSA *Shigella* spp.

| Table S1. Cont | | | | | | | | | |
| --- | --- | --- | --- | --- | --- | --- | --- | --- | --- |
|  | P50 | P68 | P72 | P76 | P77 | P79 | P82 | P85 | P86 |
| Age | Under 5 years | 6-10 years | 6-10 years | 6-10 years | 6-10 years | Under 5 years | Under 5 years | 11-14 years | 6-10 years |
| Gender | Male | Male | Male | Male | Female | Female | Male | Male | Female |
| Ward | Central lab | Emergency | OPD | OPD | OPD | Surgery | OPD | OPD | OPD |
| Resistance pattern | CTX/AMP/SXT/CFM/AZM | CTX/NA/AMP/SXT/CFM/AZM | NA/SXT/CFM/AZM/MN | CTX/NA/AMP/SXT/CFM/AZM/MN | CTX/AMP/SXT/CFM/AZM/MN | CTX/NA/AMP/SXT/CFM/AZM/MN | CTX/AMP/SXT/CFM/AZM | CTX/NA/AMP/SXT/CFM/AZM/MN | CTX/NA/AMP/SXT/CFM/AZM/MN |
| *mphA* | No | No | No | No | No | No | No | No | No |
| *mphB* | No | No | No | No | No | No | No | No | No |
| *ermA* | No | No | No | No | No | No | No | No | No |
| *ermB* | No | No | No | No | No | No | No | No | No |
| *ermC* | No | No | No | No | No | No | No | No | No |
| *ermF* | No | No | No | No | No | No | No | No | No |
| *ermX* | No | No | No | No | No | No | No | No | No |
| *ermT* | No | No | No | No | No | No | No | No | No |
| *ereA* | No | No | No | No | No | No | No | No | No |
| *ereB* | No | No | No | No | No | No | No | No | No |
| *mefA* | No | No | No | No | No | No | No | No | No |
| *msrA* | No | No | No | No | No | No | No | No | No |
| Azithromycin MIC (µg/ml) in the presence of inhibitor | 32 | 256 | 32 | 64 | 32 | 64 | 32 | 32 | 64 |
| Azithromycin MIC (µg/ml) in the absence of inhibitor | 32 | 512 | 32 | 64 | 32 | 64 | 32 | 32 | 64 |

| Table S1. Cont | | | | | | | | | |
| --- | --- | --- | --- | --- | --- | --- | --- | --- | --- |
|  | P87 | P88 | P89 | P92 | P93 | P97 | P98 | P99 | P100 |
| Age | Under 5 years | Under 5 years | 11-14 years | 11-14 years | Under 5 years | Under 5 years | Under 5 years | Under 5 years | 6-10 years |
| Gender | Male | Male | Male | Male | Male | Male | Male | Female | Female |
| Ward | OPD | OPD | Emergency | PICU | OPD | OPD | Emergency | Emergency | OPD |
| Resistance pattern | CTX/NA/AMP/SXT/CFM/AZM/MN | CTX/NA/AMP/SXT/CIP/AZM/MN | NA/AMP/SXT/CIP/CFM/AZM/MN | CTX/NA/AMP/SXT/LEV/CFM/AZM/MN | CTX/NA/AMP/SXT/CFM/AZM/NA | CTX/NA/AMP/SXT/CFM/AZM/NA | CTX/NA/AMP/SXT/CFM/AZM/NA | CTX/NA/AMP/SXT/CFM/AZM/NA | CTX/NA/AMP/SXT/CFM/AZM/NA |
| *mphA* | No | No | No | No | No | No | No | No | No |
| *mphB* | No | No | No | No | No | No | No | No | No |
| *ermA* | No | No | No | No | No | No | No | No | No |
| *ermB* | No | No | No | No | No | No | No | No | No |
| *ermC* | No | No | No | No | No | No | No | No | No |
| *ermF* | No | No | No | No | No | No | No | No | No |
| *ermX* | No | No | No | No | No | No | No | No | No |
| *ermT* | No | No | No | No | No | No | No | No | No |
| *ereA* | No | No | No | No | No | No | No | No | No |
| *ereB* | No | No | No | No | No | No | No | No | No |
| *mefA* | No | No | No | No | No | No | No | No | No |
| *msrA* | No | No | No | No | No | No | No | No | No |
| Azithromycin MIC (µg/ml) in the presence of inhibitor | 64 | 512 | 32 | 16 | 32 | 64 | 64 | 64 | 32 |
| Azithromycin MIC (µg/ml) in the absence of inhibitor | 64 | 512 | 32 | 256 | 32 | 64 | 64 | 64 | 32 |

| Table S1. Cont | | | | | | | | | |
| --- | --- | --- | --- | --- | --- | --- | --- | --- | --- |
|  | P101 | P102 | P103 | P104 | P107 | P108 | P109 | P110 | P112 |
| Age | 11-14 years | Under5 years | 6-11 years | 6-11 years | 6-11 years | 6-11 years | 6-11 years | 6-11 years | 11-14 years |
| Gender | Male | Female | Female | Female | Female | Male | Female | Male | Male |
| Ward | OPD | Emergency | Emergency | Emergency | OPD | Emergency | OPD | OPD | OPD |
| Resistance pattern | CTX/NA/AMP/SXT/LEV/CFM/AZM/MN | CTX/NA/AMP/SXT/LEV/CFM/AZM/MN | CTX/NA/AMP/SXT/LEV/CFM/AZM/MN | CTX/NA/AMP/SXT/LEV/CFM/AZM/MN | CTX/NA/AMP/SXT/LEV/CFM/AZM/MN | CTX/NA/AMP/SXT/LEV/CFM/AZM/MN | CTX/NA/AMP/SXT/LEV/CFM/AZM/MN | CTX/NA/AMP/SXT/LEV/CFM/AZM/MN | CTX/NA/AMP/SXT/ CFM/AZM/MN |
| *mphA* | No | No | No | No | No | No | No | No | No |
| *mphB* | No | No | No | No | No | No | No | No | No |
| *ermA* | No | No | No | No | No | No | No | No | No |
| *ermB* | No | No | No | No | No | No | No | No | No |
| *ermC* | No | No | No | No | No | No | No | No | No |
| *ermF* | No | No | No | No | No | No | No | No | No |
| *ermX* | No | No | No | No | No | No | No | No | No |
| *ermT* | No | No | No | No | No | No | No | No | No |
| *ereA* | No | No | No | No | No | No | No | No | No |
| *ereB* | No | No | No | No | No | No | No | No | No |
| *mefA* | No | No | No | No | No | No | No | No | No |
| *msrA* | No | No | No | No | No | No | No | No | No |
| Azithromycin MIC (µg/ml) in the presence of inhibitor | 32 | 32 | 32 | 64 | 32 | 64 | 32 | 64 | 64 |
| Azithromycin MIC (µg/ml) in the absence of inhibitor | 32 | 64 | 32 | 64 | 64 | 64 | 64 | 64 | 64 |

| Table S1. Cont | | | | | | | | | |
| --- | --- | --- | --- | --- | --- | --- | --- | --- | --- |
|  | P114 | P117 | P118 | P119 | P121 | P123 | P124 | P125 | P126 |
| Age | 6-10 years | 6-10 years | 6-10 years | 11-14 years | Under 5 years | 6-10 years | Under 5 years | Under 5 years | 6-10 years |
| Gender | Female | Male | Female | Male | Male | Female | Male | Male | Male |
| Ward | OPD | OPD | OPD | OPD | PICU | OPD | OPD | Emergency | Emergency |
| Resistance pattern | CTX/NA/AMP/SXT/ CFM/AZM/MN | CTX/NA/AMP/SXT/ CFM/AZM/MN | CTX/NA/AMP/SXT/ CFM/AZM/MN | CTX/NA/AMP/SXT/ CFM/AZM/MN | CTX/NA/AMP/SXT/CFM/AZM | CTX/NA/AMP/SXT/ CFM/AZM/MN | CTX/NA/AMP/SXT/ CFM/AZM/MN | CTX/NA/AMP/SXT/ CFM/AZM/MN | CTX/NA/AMP/SXT/ CFM/AZM/MN |
| *mphA* | No | No | No | No | No | No | No | Yes | No |
| *mphB* | No | No | No | No | No | No | No | No | No |
| *ermA* | No | No | No | No | No | No | No | No | No |
| *ermB* | No | No | No | No | No | No | No | No | No |
| *ermC* | No | No | No | No | No | No | No | No | No |
| *ermF* | No | No | No | No | No | No | No | No | No |
| *ermX* | No | No | No | No | No | No | No | No | No |
| *ermT* | No | No | No | No | No | No | No | No | No |
| *ereA* | No | No | No | No | No | No | No | No | No |
| *ereB* | No | No | No | No | No | No | No | No | No |
| *mefA* | No | No | No | No | No | No | No | No | No |
| *msrA* | No | No | No | No | No | No | No | No | No |
| Azithromycin MIC (µg/ml) in the presence of inhibitor | 64 | 64 | 64 | 64 | 32 | 64 | 32 | 32 | 32 |
| Azithromycin MIC (µg/ml) in the absence of inhibitor | 64 | 64 | 64 | 64 | 32 | 64 | 32 | 32 | 64 |

| Table S1. Cont | | | | | | | | | |
| --- | --- | --- | --- | --- | --- | --- | --- | --- | --- |
|  | P127 | P128 | P130 | P133 | P134 | P135 | P136 | P143 | P145 |
| Age | Under 5 years | Under 5 years | 6-10 years | Under 5 years | Under 5 years | 6-10 years | Under 5 years | 6-10 years | 6-10 years |
| Gender | Male | Male | Male | Male | Female | Female | Male | Male | Female |
| Ward | Emergency | OPD | Emergency | OPD | Infant ICU | OPD | Emergency | OPD | OPD |
| Resistance pattern | CTX/NA/AMP/SXT/ CFM/AZM/MN | CTX/NA/AMP/SXT/ CFM/AZM/MN | CTX/NA/AMP/SXT/ CFM/AZM/MN | CTX/NA/AMP/SXT/ CFM/AZM/MN | CTX/NA/AMP/SXT/ CFM/AZM/MN | CTX/NA/AMP/SXT/ CFM/AZM/MN | CTX/NA/AMP/SXT/ CFM/AZM/MN | CTX/NA/AMP/SXT/AZM | CTX/NA/AMP/SXT/ CFM/AZM/MN |
| *mphA* | No | No | No | No | No | No | No | No | No |
| *mphB* | No | No | No | No | No | No | No | No | No |
| *ermA* | No | No | No | No | No | No | No | No | No |
| *ermB* | No | No | No | No | No | No | No | No | No |
| *ermC* | No | No | No | No | No | No | No | No | No |
| *ermF* | No | No | No | No | No | No | No | No | No |
| *ermX* | No | No | No | No | No | No | No | No | No |
| *ermT* | No | No | No | No | No | No | No | No | No |
| *ereA* | No | No | No | No | No | No | No | No | No |
| *ereB* | No | No | No | No | No | No | No | No | No |
| *mefA* | No | No | No | No | No | No | No | No | No |
| *msrA* | No | No | No | No | No | No | No | No | No |
| Azithromycin MIC (µg/ml) in the presence of inhibitor | 32 | 64 | 32 | 64 | 64 | 32 | 32 | 32 | 64 |
| Azithromycin MIC (µg/ml) in the absence of inhibitor | 64 | 64 | 32 | 64 | 64 | 32 | 32 | 32 | 64 |
